# Hand and Foot Glomus Tumors: Significance of MRI Diagnosis Followed by Histopathological Assessment

**DOI:** 10.7759/cureus.30038

**Published:** 2022-10-07

**Authors:** Tarang Patel, Virendrakumar Meena, Pinki Meena

**Affiliations:** 1 Pathology, All India Institute of Medical Sciences (AIIMS), Rajkot, IND; 2 Radiology, Geetanjali Medical College & Hospital, Udaipur, IND; 3 Anaesthesiology, Geetanjali Medical College & Hospital, Udaipur, IND

**Keywords:** histopathological examination, surgical excision, subungual location, mri imaging, glomus tumour

## Abstract

Introduction

Glomus tumors are benign lesions with hamartomatous proliferation in the neuromyoarterial glomus bodies. Glomus tumors are small, reddish, painful blue nodules usually underneath the fingernail.

Objectives

This study is mainly focusing on magnetic resonance imaging (MRI) findings of glomus tumors on T1, T2, short inversion time inversion recovery (STIR), and post-gadolinium images. Further study of clinical and histopathological findings to support the radiological diagnosis.

Material & methods

The retrospective study included an elaborate study of MRI imaging findings of 24 cases of glomus tumors of the hand and leg at a tertiary care center in Udaipur. Patients with imaging findings confirmed on histopathology were included in the study. MRI study was conducted using a 3-T MR unit and a high-spatial-resolution module.

Results

A total of 24 cases of glomus tumors were diagnosed during the six years between January 2015 and November 2020. Out of 24 patients, 14 were female and 10 were male. The most common site of involvement was a hand, followed by a foot. All 24 cases showed isointense to hyperintense lesions on T2-weighted images with a conspicuous hyperintense lesion on STIR images. Further histopathological examination confirmed the diagnosis showing nests of monomorphic tumor nuclei arranged in a perivascular pattern.

Conclusion

Glomus tumors can present with variable pain. A high index of suspicion is needed for diagnosis. Contrast MRI has a significant role in the diagnosis of glomus tumors. The clinical and histopathological picture further confirms the diagnosis. MRI imaging is further supportive to differentiate postoperative fibrosis from residual or recurrent tumors.

## Introduction

Glomus bodies are present in the reticular layer of the dermis of the whole body but are numerously seen in digits, feet soles, and hand palms. These bodies have arteriovenous anastomoses, which are very helpful in thermoregulation by the regulation of skin blood flow [1]. Glomus tumor was first described by Wood in 1812 as benign lesions consisting of hamartomatous proliferation in the neuromyoarterial glomus bodies [2-4]. However, in 1924, Masson described the histopathological findings and gave the ‘Glomus’ name. That's why these tumors are also known as Barre-Masson syndrome [2,5,6].

These tumors are also known as glomangiomas and represent approximately 2% of all primary soft tissue tumors and 1% to 4.5% of hand neoplasms [7,8]. Glomus tumors can be solitary or multiple. The solitary type is usually present in digits [9]. The etiology of glomus tumors is largely unknown, and it may be related to gender, age, or trauma. Trauma can cause reactive hypertrophy in the structure of a glomus body. There is a familial variant of glomus tumor that had been linked to chromosome 1p21-22 and involved truncating mutations in the glomulin gene [10].

Glomus tumors are small and reddish-blue nodules measuring 3 to 10 mm in diameter [2]. The subungual region of distal phalanges is a typical location for these tumors but may be present throughout the body. The patient usually presents with a small firm nodule underneath the fingernail with intense pain and is very sensitive to pressure and temperature. Pain is believed to be due to stretching of the capsule and mast cell releasing substances while some owe it to a network of non-myelinated nerve fibers that penetrate glomus tumors. Changes in the morphology of the ungual region can occur if lesions are larger and, very rarely, these tumors are palpable [11,12]. Because the clinical signs are not always obvious, imaging is very helpful in diagnosis and further treatment planning [13,14]. This study is mainly focusing on the role of MRI imaging findings of subungual glomus tumors in correlation with clinical and histopathological findings.

## Materials and methods

A retrospective study of 24 cases of glomus tumors showing typical findings on MRI imaging, was carried out for the study period between January 2015 to November 2020 at a tertiary care center in Udaipur, Rajasthan. Medical records were retrieved and reviewed for demographics, symptoms, duration, physical examination, and recurrence.

Inclusion criteria

The following patients were included: 1. Patients with typical MRI features of glomus tumors of the hand and foot; 2. Patients admitted to our hospital and operated on for surgical excision; 3. Diagnosis of glomus tumor confirmed on histopathological examination.

Exclusion criteria

Patients without histopathological confirmation of glomus tumors or glomus tumors other than the hand and foot were excluded from the study.

MRI protocol

All MR imaging examinations were performed with a 3-T MR unit (Signa Architect; GE Medical Systems, Milwaukee, Wis). A high-spatial-resolution module for skin imaging was used in all patients. A surface gradient coil was used in place of the anteroposterior gradient coil of the imaging system to increase the gradient strengths. A surface radio-frequency coil with a 1.5-cm radius was placed at the center of the gradient coil. All MR images were retrospectively reviewed in consensus by two radiologists with more than four years of experience who were blinded to the surgical findings.

The patterns of glomus tumors were also noted. The location of each tumor in the sagittal plane and transverse plane was noted. The margins of the tumor were classified and the largest diameter of the tumor was measured in millimeters in the transverse and sagittal planes. The signal intensity of each tumor on T1- and T2-weighted spin-echo MR images compared with the signal intensity of the normal nail bed was characterized as low, equivalent (isointense), or high and as either homogeneous or heterogeneous. The appearance of the tumor after intravenous injection of gadolinium was characterized as no enhancement, slight enhancement, and intense enhancement compared with the appearance of the nail bed. On MR angiograms, an early nodular enhancement in the arterial phase was considered to be diagnostic of a glomus tumor [15].

For excising subungual lesions trans-ungual approach, lateral or dorsal approaches were used and those with pulp lesions were excised with a direct incision over the affected site. All specimens were subjected to a histopathological study. Histopathological examination was carried out on formalin-fixed paraffin-embedded sections. Sutures were removed after 14 days.

## Results

A total of 379 MRIs for hand and foot were done in the last five years. Sixty-seven MRIs were done for pain in the fingers and toes, out of which 24 patients were found to have features of glomus tumors. These 24 patients were referred from the orthopedics and dermatology department. Out of 24, 14 were female and 10 were male.

Age ranges from 19 to 68 years with a mean age was 35.8 ± 8.5 years. The most common anatomical site of the tumor was the hand (19 cases) followed by the foot (five cases). The range of duration between clinical symptoms and diagnosis was four to 62 months and the mean duration was 16.9 ± 13.2 months.

The left hand was affected in 11 cases while the right hand was affected in eight cases. The thumb, index, and middle finger were involved in four cases, eight cases, and six cases respectively, while one case involved the little finger. The right great toe was affected in three cases while two cases occurred in the left great toe.

Fourteen patients complained of digit pain, and it was localized to the terminal phalanx of the finger. Three patients had pain localized to the great toe. Fourteen patients clinically presented with swelling at the site of pain. Cold hypersensitivity and night aggravation of pain were positive for eight patients. Love’s pin test (pinpoint tenderness) localized the lesion in all 24 cases (100%). Hildreth’s sign was positive in six out of the eight cases where it was done (75%). The pain was reproducible on palpation in all 24 hand and foot cases.

**Figure 1:**
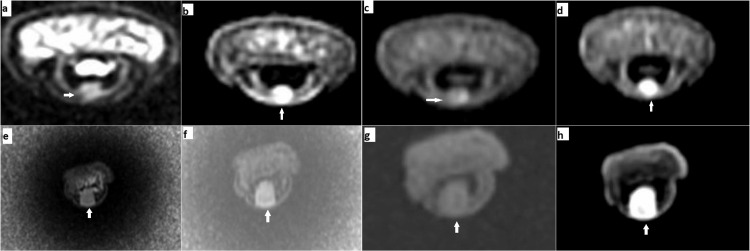
MRI findings of the index and little fingers (a,b,c,d - index finger; e,f,g,h - little finger). Axial T2-weighted image shows an iso-intense lesion in the distal phalanx of the index finger (a) and distal phalanx of the little finger (e). Short inversion time inversion recovery (STIR) images suggest the lesion was more conspicuous (b,f). Hypo-intense on T1-weighted fat-saturated image (c,g). T1 post-gadolinium fat saturation images show avid homogenous post-contrast enhancement (d,h).

The X-ray imaging and routine laboratory test were within normal limits. All 24 patients underwent contrast-enhanced magnetic resonance imaging (CEMRI). All of them revealed isointense to hyperintense lesions on T2. Twenty-three patients revealed intense contrast enhancement on T1 post-gadolinium fat saturation images, and one patient showed minimal contrast enhancement. One male and one female patient revealed well-defined, spherical, T2-weighted, iso-intense lesions in the subungual aspect of the index finger and little finger, respectively. Both lesions were more conspicuous on short inversion time inversion recovery (STIR) images and hypo-intense on T1-weighted fat-saturated image with avid homogenous post-contrast enhancement was seen on T1 post-gadolinium fat saturation images (Figures 1a-1h).

**Figure 2:**
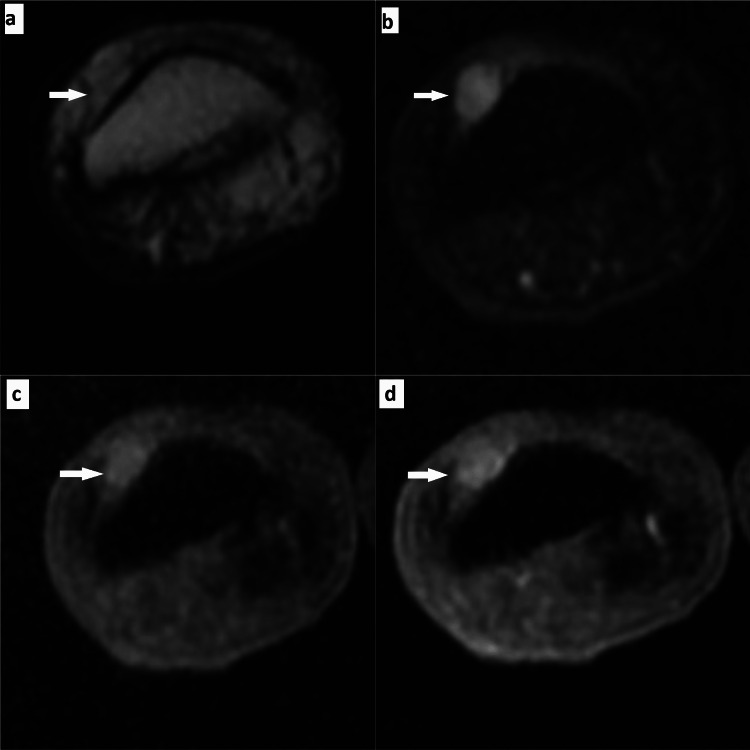
MRI findings of a great toe lesion Axial T2-weighted image shows an iso-intense lesion in the subungual aspect of the great toe (a). Short inversion time inversion recovery (STIR) images suggest the lesion was more conspicuous (b) Hypo-intense on a T1-weighted, fat-saturated image (c). Minimal to mild post-contrast enhancement was seen on T1 post-gadolinium fat saturation images (d).

One female patient present with a complaint of toe pain revealed a well-defined, spherical, T2-weighted iso-intense lesion in the subungual aspect of the great toe. The lesion was more conspicuous on STIR images and hypo-intense on a T1-weighted, fat-saturated image with minimal post-contrast enhancement seen on T1 post-gadolinium fat saturation images (Figure 2).

**Figure 3:**
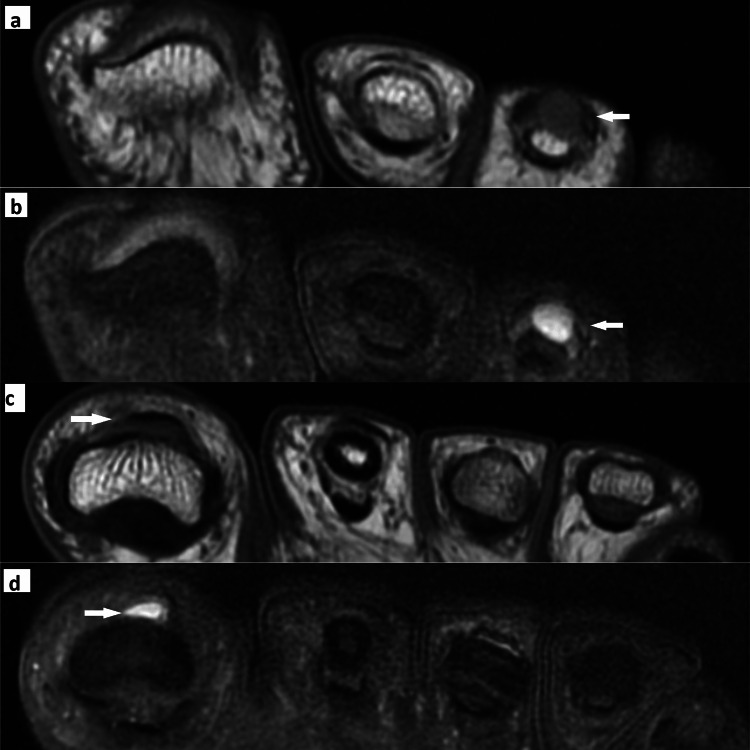
MRI features of the third toe swelling Axial T1-weighted image (a) shows an isointense lesion in the subungual aspect of the third toe and it appears hyperintense on the axial T2 fat-saturated (FS) image (b). The same patient's axial T1-weighted image (c) shows an isointense lesion in the subungual aspect of the great toe, and it appears hyperintense on the axial T2 FS image (d)

One female presented with two separate glomus tumors on the great toe and third toe of the left foot and both tumors appear isointense on the T1-weighted image and hyperintense on the STIR image (Figure 3).

**Figure 4:**
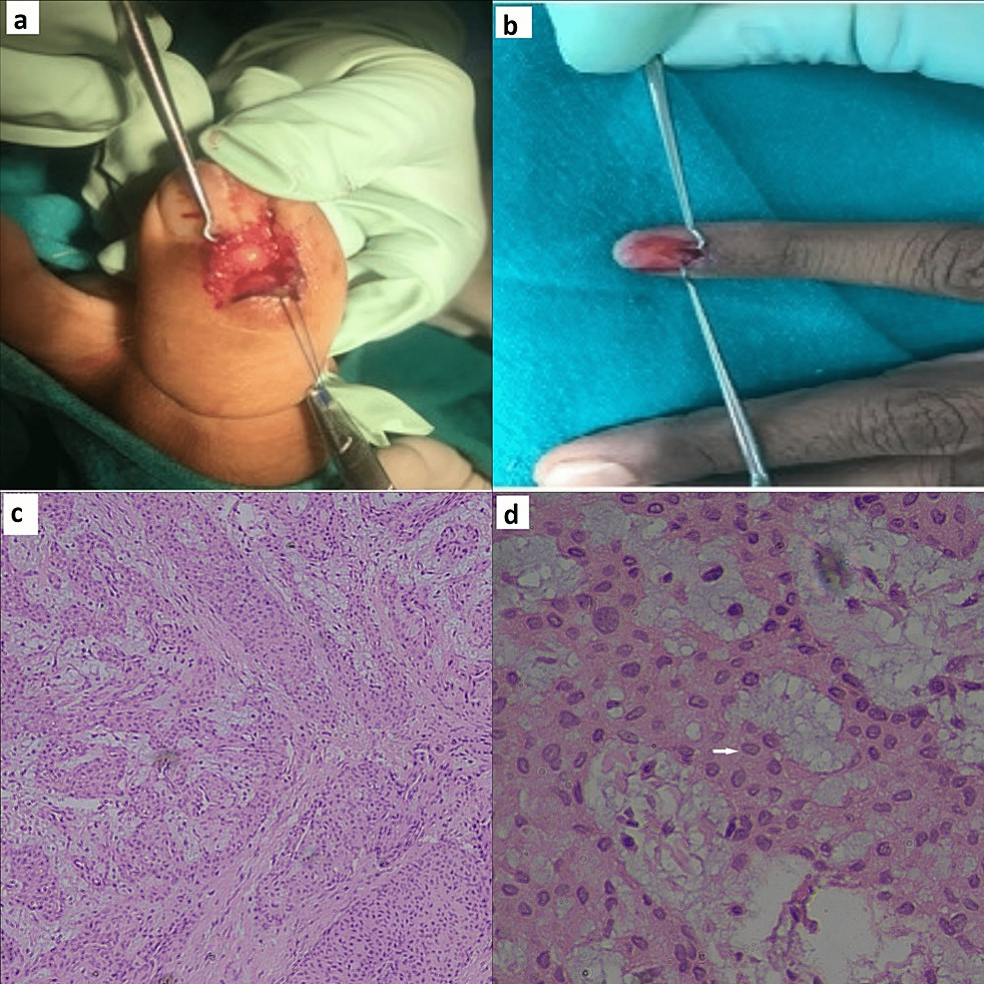
Gross and microscopic findings (a,b) Glomus tumor of the great toe and index finger during exploration. (c) A tumor composed of sheets and a nest of uniform round cells, interrupted by vessels of varying size (H&E, 4x). (d) High-power examination revealed that the glomus tumor cells exhibited relatively uniform round to oval nuclei (arrow) and pale cytoplasm (H&E, 40x).

Surgical excision was performed, which revealed well-defined brown masses with no adjacent soft tissue extensions (Figures 4a-4b). The resulting histopathology was consistent with the preoperative diagnosis of a glomus tumor. Histopathology images show a tumor composed of sheets and a nest of uniform round cells, interrupted by vessels of varying sizes. High-power examination revealed that the glomus tumor cells exhibited punched-out round to oval nuclei and pale cytoplasm (Figures 4c-4d) while one case that did not have avid contrast turned out to be a myxoid form of glomus tumor.

## Discussion

A glomus tumor is a rare benign vascular tumor of the neuromyoarterial glomus body. About 80% of cases are found on the upper limbs, mostly under the nails [16].

The normal subungual space is a small, space of 1-2 mm below the nail. Therefore, tumors of this space, irrespective of their histologic nature are painful and often erode into the distal phalanx of the digits. Tumors that affect this space include majorly benign solid tumors like glomus tumors, subungual exostosis, soft-tissue chondroma, keratoacanthoma, hemangioma, lobular capillary hemangioma, and benign cystic lesions like epidermal and mucoid cysts. Though there are some malignant tumors like squamous cell carcinoma and malignant melanoma that are also seen in subungual space [17,18].

Various literature has reported a fairly long duration from clinical symptoms to diagnosis [19]. This is probably because in that study USG was the primary modality of diagnosis as compared to contrast-enhanced MRI [20]. Yilmaz et al. reported an average delay of seven years and four months. They found that this delay was due to misdiagnosis and due to treatment with nonsteroidal anti-inflammatory drugs for pain [6].

The characteristics of glomus tumors are the triad of localized pain, cold sensitivity, and point tenderness [21]. Van Geertruyden et al. described pain, mild tenderness, and cold sensitivity in 80%, 100%, and 63%. respectively, in their series of 51 patients [22].

One study of 24 cases of hand glomus tumor recorded the sensitivity and specificity of Hildreth’s test to be 92% and 91%, respectively [23]. However, in this present study, we did not involve Hildreth's test. Another study found cold sensitivity as 100% sensitive, specific and accurate, whereas Love’s pin test as 100% sensitive and 78% accurate [24].

Sometimes, it is very difficult to make the correct diagnosis of glomus tumors on the clinical background. Despite many clinical tests for this tumor, glomus tumors may still go undetected because of the small size of the lesion, which may delay the diagnosis. So radiological imaging plays the principal role in its diagnosis [25].

Recent studies favor MRI and ultrasound as the preferred radiological methods to identify a glomus tumor [26]. Plain radiographs are not useful in the detection of glomus tumors without any adjacent bony erosion in the late stage of the disease [27]. Ultrasound and Doppler are also helpful in the detection of glomus tumors but because of the small size of the lesion, operator dependency, and difficulty in sonographic interpretation of subungual region, ultrasound is not a good imaging modality in a case of strong clinical suspicion of glomus tumor. Apart from that curvature of the nail plate may create artifacts in the lateral nail folds in ultrasonography [6,14,20]. MRI is the most sensitive imaging modality for the early detection of glomus tumors. They are well-defined nodular lesions in the subungual region with hypo-intense signal intensity on the T1-weighted sequence, hyper-intense signal intensity on the T2-weighted sequence, and post-contrast T1W fat-suppressed sequence showing intense homogenous contrast enhancement [1,15].

These classic MRI findings are not specific to glomus tumors and can also be seen with cysts and other solid hand tumors. Post-contrast gadolinium-enhanced, T1W fat-suppressed (FS) sequence shows intense homogenous contrast enhancement can help differentiate glomus tumors from mucoid cysts and epithelial inclusion cysts because the latter two do not enhance. Most glomus tumors contain mainly three components and the components are vessels, glomus cells, and mucoid tissue, so the vascular form shows strong enhancement while solid and mucoid form shows less enhancement [2].

MRI provides sufficient information about the extent and location of the tumor and is very helpful in the planning of surgery. Complete surgical removal of tumors is the treatment of choice for glomus tumors. The incidence of postoperative recurrence is 12-24% of cases. If recurrence of the symptom is within less than one year of surgery then incomplete resection is the likely cause and if recurrence is after more than one year then the development of the new lesion is the likely cause [1,2]. Imaging helps recognize bony invasion in clinically obvious large lesions, which is again an important factor in the decision of surgical excision [28].

Histopathology shows subcutaneous proliferation of well-circumscribed tumor nodules showing cuffs of glomus cells surrounding thin-walled blood vessels. Glomus cells show small, uniform round nuclei with amphophilic to eosinophilic cytoplasm and well-defined cell borders. Histopathological variants of Glomus tumor are oncocytic, epithelioid, myxoid, and hyalinized. A glomus tumor containing spindle cells and branching vasculature is known as ‘glomangiomyoma’ and ‘glomangiopericytoma’, respectively. A ‘symplastic glomus tumor’ is reserved for marked nuclear atypia without increased mitosis. A malignant glomus tumor is diagnosed in the presence of significant nuclear atypia and increased mitosis, including atypical ones [29].

MRI is very helpful in the differentiation of recurrent/ residual tumors with postoperative fibrotic changes. Postoperative changes are usually ill-defined and appear hypo-intense on all sequences, whereas recurrence and residual lesions show similar findings as that of a non-treated lesion [1]. Mucoid and epidermoid inclusion cysts are considered a differential diagnosis of glomus tumors but show poor contrast enhancement after intravenous gadolinium administration [11].

## Conclusions

A glomus tumor is a rare tumor and its differential diagnosis should be kept in mind while assessing a patient with pain and swelling of a fingertip. Complete surgical excision is enough for the treatment of glomus tumors, with a very low incidence of recurrence.

MRI imaging along with clinic-pathological findings have a very significant role in the precise and early diagnosis of glomus tumors. Apart from diagnosis, MRI is also very helpful in identifying the exact location and extent of the lesion and ruling out a postoperative residual tumor. Some rare solid and mucoid variants are difficult to confirm on MRI. A combined patho-radiological approach is demanded in such cases. Histopathological confirmation is essential to describe any variant of glomus tumors and to rule out features indicating their malignant nature.

## References

[1] Theumann NH, Goettmann S, Le Viet D (2002). Recurrent glomus tumors of fingertips: MR imaging evaluation. Radiology.

[2] Drapé JL, Idy-Peretti I, Goettmann S (1995). Subungual glomus tumors: evaluation with MR imaging. Radiology.

[3] Vanti AA, Cuce LC, Di Chiacchio N (2007). Subungual glomus tumor: epidemiological and retrospective study, from 1991 to 2003. An Bras Dermatol.

[4] Wood W (1812). On painful subcutaneous tubercle. Edinb Med Surg J.

[5] Masson P (1924). Le glomus neuromyoarterial des regions textiles et ses tumeurs [Article in French]. Lyon Chir.

[6] Tomak Y, Akcay I, Dabak N, Eroglu L (2003). Subungual glomus tumours of the hand: diagnosis and treatment of 14 cases. Scand J Plast Reconstr Surg Hand Surg.

[7] Carroll RE, Berman AT (1972). Glomus tumors of the hand: review of the literature and report on twenty-eight cases. J Bone Joint Surg Am.

[8] Fornage BD (1988). Glomus tumors in the fingers: diagnosis with US. Radiology.

[9] Tang CY, Tipoe T, Fung B (2013). Where is the lesion. Glomus tumours of the hand?. Arch Plast Surg.

[10] Brouillard P, Boon LM, Mulliken JB (2002). Mutations in a novel factor, glomulin, are responsible for glomuvenous malformations ("glomangiomas"). Am J Hum Genet.

[11] Morey VM, Garg B, Kotwal PP (2016). Glomus tumours of the hand: review of literature. J Clin Orthop Trauma.

[12] Rodríguez JM, Idoate MA, Pardo-Mindán FJ (2003). The role of mast cells in glomus tumours: report of a case of an intramuscular glomus tumour with a prominent mastocytic component. Histopathology.

[13] Kale SS, Rao VK, Bentz ML (2006). Glomus tumor of the index finger. J Craniofac Surg.

[14] Chen SH, Chen YL, Cheng MH, Yeow KM, Chen HC, Wei FC (2003). The use of ultrasonography in preoperative localization of digital glomus tumors. Plast Reconstr Surg.

[15] Al-Qattan MM, Al-Namla A, Al-Thunayan A, Al-Subhi F, El-Shayeb AF (2005). Magnetic resonance imaging in the diagnosis of glomus tumours of the hand. J Hand Surg Br.

[16] Samaniego E, Crespo A, Sanz A (2009). Key diagnostic features and treatment of subungual glomus tumor [Article in Spanish]. Actas Dermosifiliogr.

[17] Haneke E (2006). Surgical anatomy of the nail apparatus. Dermatol Clin.

[18] Teh J, Whiteley G (2007). MRI of soft tissue masses of the hand and wrist. Br J Radiol.

[19] Pandey CR, Singh N, Tamang B (2017). Subungual glomus tumours: Is magnetic resonance imaging or ultrasound necessary for diagnosis?. Malays Orthop J.

[20] Santoshi JA, Kori VK, Khurana U (2019). Glomus tumor of the fingertips: a frequently missed diagnosis. J Family Med Prim Care.

[21] Ham KW, Yun IS, Tark KC (2013). Glomus tumors: symptom variations and magnetic resonance imaging for diagnosis. Arch Plast Surg.

[22] Van Geertruyden J, Lorea P, Goldschmidt D (1996). Glomus tumours of the hand. A retrospective study of 51 cases. J Hand Surg Br.

[23] Giele H (2002). Hildreth's test is a reliable clinical sign for the diagnosis of glomus tumours. J Hand Surg Br.

[24] Netscher DT, Aburto J, Koepplinger M (2012). Subungual glomus tumor. J Hand Surg Am.

[25] Dinesh E, Terence T, Ruben J (2011). Subungual glomus tumour: magnetic resonance imaging and treatment: a case report. Malays Orthop J.

[26] Kim DH (1999). Glomus tumour of the fingertip and MRI appearance. Iowa Orthop J.

[27] Lee W, Kwon SB, Cho SH, Eo SR, Kwon C (2015). Glomus tumor of the hand. Arch Plast Surg.

[28] Aboueldahab AK, Elkafrawi H, Ghozlan NA (2011). Glomus tumour of the fingers: accurate preoperative localization as a prerequisite to avoid tumour recurrence. Egypt J Plast Reconstr Surg.

[29] Mravic M, LaChaud G, Nguyen A, Scott MA, Dry SM, James AW (2015). Clinical and histopathological diagnosis of glomus tumor: an institutional experience of 138 cases. Int J Surg Pathol.

